# The Hospital World

**Published:** 1910-11-12

**Authors:** 


### The Hospital World.

### THE LATE MR. HENRY LUCAS.

MR. HENRY LUCAS, the well-known treasurer of University College Hospital, died on November 5 at his country house near East Grinstead. The funeral took place at the Jewish Cemetery, Willesden, on Tuesday last. In him University College Hospital had an old and tried friend who was never more devoted to its interests than in times of crisis, and whose experience of its needs had extended over a period of twenty-seven years. We may note in passing that he was educated at University College, was called to the Bar at Lincoln's Inn in 1869, and practised as a conveyancer till 1891, when he retired. It is no exaggeration to say, however, that his chief interests were public and philanthropic, and his first official connection with the hospital to which he devoted afterwards so much of his energy took place with his appointment as member of the General Committee in 1883. He was at one time Vice-President of the United Synagogue and President of the Jewish Religious Education Board. Nine years later, in 1892 in fact, he became its Chairman; and perhaps this year especially may be said to mark the second and most intimate period of his work for University College Hospital. For it was in 1893 that the Rebuilding Committee began to face the mighty task of reconstruction, and Mr. Lucas's courage and energy in the face of difficulties which would have dismayed many men were simply invaluable. We have only to remind our readers that the work of the Rebuilding Committee, which began, as said above, in 1893, was not completed till 1905, when the University College, London (Transfer), Act for the incorporation of the hospital was passed. During this strenuous period, moreover, other labours for the hospital engaged him. In 1898 Mr. Lucas received his late Majesty, then Prince of Wales, when the foundation-stone of the new hospital was laid by him. As though the peculiar intricacies of a Building Committee's problems had a special attraction for his mind, we find Mr. Lucas in 1904 sitting as chairman of another committee, the work of which comprised such important questions as the rebuilding of the medical School, of the Nurses' Institute, and Students' House. The crown was placed upon these labours for him in that triumphant year 1906, when the Duke of Connaught came in person to open the new hospital. The reconstruction finished, the buildings complete, the inevitable problem of providing an income to maintain them in full working order then had to be faced. Mr. Lucas, who had worked so hard at the former, again threw his energies into the field. Plans of bricks and mortar and contractors' estimates gave place on his table to hospital accounts and appeals. In 1907 he was appointed Treasurer of the hospital. To his work in that capacity he devoted himself to the day of his death, and his gifts amounting to over £1,000, in addition to the bed endowed jointly by Mrs. Lucas, daughter of the late Mr. N. M. Montefiore, and himself in a sum of £2,000, show that he was the apostle of his own enthusiasm. It is no slight on posterity to say that his place will be hard to fill, but his example will remain to show that great difficulties, at University College Hospital at any rate, have a way of producing the men capable to meet them. It must be with this feeling that the hospital faces its future and its loss in him to-day.

### Personalia.

ON Wednesday evening, November 2, the following address was presented to Sir Vezey Strong, J.P., Chairman of the board of management of the London Temperance Hospital, and Lord Mayor-Elect of the City of London :

"May we, speaking for ourselves as well as on behalf of all others interested in the London Temperance Hospital, warmly congratulate you on your election to the high and honourable office of Lord Mayor of London? The loyal and devoted service so long rendered by you to the cause of philanthropy is widely known and highly appreciated, but by none more than by your colleagues in connection with the Temperance Hospital, with whom you have been associated as a member of the board of management, Vice-Chairman and Chairman, during a period of twenty years. Your kindness and courtesy, your wisdom in counsel, and your firmness in execution have contributed to the success of the hospital in a remarkable degree; your sound and skilful advice in the control of the funds of the hospital have elicited the cordial and special thanks of the board of management, and you have earned the admiration and affectionate esteem of all with whom you have been associated. As Lord Mayor of London you will fill one of the most prominent offices open to citizens of the British Empire, an office honourably and continuously maintained for seven centuries. We rejoice that so important an office should be occupied by a man of your sterling principle, one worthy in every way to maintain the high traditions of the mayoralty. We pray that God may bestow upon you and Lady Strong His richest blessings, that He may grant you health and strength adequate for your arduous duties, and that He may continue for many years to make your lives fruitful in every good work."

The address was presented by Lord Alverstone, as President of the hospital, and was signed by him, by the members of the board, and by members of the medical and nursing staffs. Lord Alverstone, in presenting the address, said that, whilst his previous visits to the hospital had given him pleasure, no visit had caused him greater pleasure than the occasion of the presentation of this address. He expressed his satisfaction at the manner in which Sir Vezey had striven to conduct the affairs of the hospital with which he had been so long associated, and assured him of the continued support of all present. Sir Vezey, in reply, expressed his deep sense of gratitude for the great kindness displayed to him, and recalled his long association with the hospital, which dated from the laying of the foundation-stone. He added that, although at that time his association was not official, the reports of that date showed that he was even then a humble contributor to its funds. He would ever remember this occasion and cherish the friendship indicated in the address. A vote of thanks to Lord Alverstone for making the presentation was proposed, on behalf of the signatories, by Colonel Bolland, Vice-Chairman, seconded by Sir Edward Fithian, supported by Dr. Porter Parkinson, and carried with great enthusiasm.

AT a meeting of the Town Council of the Borough of Leicester, held on Wednesday, Alderman W. W. Vincent was elected Mayor for the ensuing year. Alderman Vincent has performed eminent services for the borough. He is Chairman of the Finance Committee, Vice-Chairman of the Derwent Valley Water Board, and a Justice of the Peace both for the county and borough. He filled the mayoral chair in 1902, during which time he prominently associated himself with Sir Edward Wood in the introduction of an extended Hospital Saturday movement in the town. All philanthropic and charitable effort in the borough has had the generous support of Alderman Vincent. He has for many years been chairman of the Finance Committee of the infirmary, a life governor of the institution, and a liberal contributor to its funds. Coronation year will be a year of activity and general rejoicing, and great satisfaction is expressed that Alderman Vincent, as Mayor, will direct the local festivities and the Commemoration movement. Miss Vincent will carry out the duties of Mayoress, which she performed during her father's previous mayoralty.

